# Same but Different? Exploring the Role of Patient-Reported Outcome Measures and Clinician-Reported Outcome Measures in Postoperative Knee and Hip Arthroplasty Rehabilitation

**DOI:** 10.3390/jcm14072322

**Published:** 2025-03-28

**Authors:** Alexandra Unger, Ferdinand Prüfer, Špela Matko, Michael J. Fischer, Vincent Grote

**Affiliations:** 1 Ludwig Boltzmann Institute for Rehabilitation Research, 1140 Vienna, Austria; 2 University of Teacher Education, 9020 Klagenfurt, Austria; 3 Rehabilitation Center Kitzbühel, 6370 Kitzbühel, Austria

**Keywords:** outcome measurement, measurement properties, responsiveness, performance score, orthopaedic rehabilitation

## Abstract

**Background/Objectives**: Patient-reported outcome measures (PROMs) and clinician-reported outcome measures (CROMs) are used in orthopaedic rehabilitation to evaluate patients’ health status and recovery. However, controversy still exists regarding their relevance and validity. This evaluation was conducted to assess the effectiveness and role of PROMs and CROMs in the orthopaedic rehabilitation outcome of patients who have undergone either total knee arthroplasty or hip arthroplasty. **Methods**: Outcome measures of 409 patients (68.3 ± 9.3 years; 34.2% male) with total knee arthroplasty and 308 patients (68.1 ± 10.6 years; 36.3% male) with total hip arthroplasty (control group) were assessed at baseline and after 21 days of inpatient rehabilitation. Effect sizes and correlations were analysed as they related to the use of PROMs and CROMs. To reduce statistical distortions caused by ceiling effects, we used a performance score (T2D) relying on two scores taken at the beginning and end of rehabilitation. **Results**: Large effect sizes (*d* > 0.97) for CROMs and medium effect sizes (*d* ≥ 0.65) for PROMs were observed in both groups. The standardised mean difference across all outcomes was 0.83 ± 0.59. PROMs worsened in 13.1% of all patients, while almost no patients showed a deterioration in CROMs. Correlations were highest between the Timed Up and Go and the Health Assessment Questionnaire scores (ρ > 0.45). **Conclusions**: Different and complementary assessment modalities of PROMs and CROMs serve as valuable clinical tools, providing a valid basis for interpreting patients’ health outcomes.

## 1. Introduction

Given the rising prevalence of hip and knee arthroplasties [1], ensuring the effectiveness of rehabilitation has become crucial. Consequently, outcome assessment plays a pivotal role in determining the most effective and efficient rehabilitation strategies to optimise recovery and functional outcomes. Different methods are used to evaluate the success of rehabilitation programmes, monitor treatment progress, and record patients’ impressions or expectations. Clinically based, performance-based, observer-reported, and patient-reported outcome measures are frequently used [2,3]. However, in joint replacement studies, outcome measures show high levels of variability due to the different scoring systems and heterogeneous reporting methods [4,5,6,7,8].

For a long time, the focus of outcome measures was predominantly directed towards clinical parameters recorded by conducting laboratory tests or performance measurements, summarised as clinician-reported outcome measures (CROMs) [9]. Following knee or hip arthroplasty, these typically include range of motion (ROM), stability, complication rates, and radiological parameters [10,11,12,13,14]. ROM measurements are carried out using goniometers, smartphone apps, or radiological joint angle determination [15,16]. Performance-based tests such as the 30 s chair-stand test, the 40-metre fast walk test, and the Timed Up and Go Test (TUG) are used for functional assessment [17]. The TUG measures the time it takes a person to stand up from a chair, reach a line three metres away, turn, and return to their starting position [18]. This test quantifies performance with tasks mimicking activities of daily living; the 6 min walk (6 MW) or the stair-climbing test (SCT) are also reliable performance tests that provide information about functional capacity in everyday situations [19].

Patient-reported outcome measures (PROMs) record subjective complaints and functional limitations and supplement clinical findings in patient-centred care [20,21]. PROMs may be useful for screening (e.g., to identify hidden problems), monitoring (e.g., to overview the effectiveness of therapies), strengthening patient-centred care (e.g., to achieve a higher patient compliance), and assessing the quality of care (e.g., to discover the strengths and weakness of therapies) [3]. These measures can be categorised as either generic or specific instruments. Generic measures provide a broad health quality assessment for different diseases and populations [22]. The Health Assessment Questionnaire (HAQ) measures disability, pain, medication effects, cost of care, and mortality [23]. The EQ-5D assesses mobility, self-care, daily activities, pain/discomfort, and anxiety/depression [24]. Visual analogue scales (VAS), verbal rating scales (VRS), and numerical pain scales (NPRS) are used to assess subjective pain [25,26]. Specific PROMs allow for a detailed assessment of specific conditions [27]. The Western Ontario and McMaster Universities Osteoarthritis Index (WOMAC) was developed to evaluate pain, stiffness, and function in people with knee and hip osteoarthritis [2,3,28,29,30].

Despite the potential for both PROMs and CROMs to demonstrate characteristic changes during rehabilitation, studies have demonstrated only a modest correlation between them [5,13,14,31,32,33,34,35,36], typically ranging from 0.4 to 0.6 [36,37,38]. This finding suggests that PROMs and CROMs may reflect distinct aspects of recovery. Fluctuations in the results of PROMs and CROMs can occur independently of each other and lead to a discrepancy between subjectively perceived and objectively measured recovery. This discrepancy can have clinical implications, as inadequate recognition of patients’ subjective complaints or functional improvements makes it difficult to accurately assess treatment success. This can lead to impairment of the adaptation of therapy to specific rehabilitation needs. Despite the relevance of this topic, recent studies have often considered PROMs and CROMs in isolation without systematically investigating their complementary or differentiating functions [9,11,13,21]. An in-depth understanding of the synergistic potential of an integrated assessment has not yet been comprehensively established. Furthermore, current clinical guidelines lack clear recommendations on the weighting and prioritisation of the results of PROMs and CROMs in the context of primary and secondary outcome measures. It is therefore crucial to precisely define these interrelationships in order to develop evidence-based guidelines for optimised and individualised rehabilitation assessment.

The aim of this study was to investigate the correlation and changes in PROMs and CROMs in patients undergoing total knee arthroplasty and compare these with the outcomes of patients undergoing total hip arthroplasty. By comparing these outcome measures across groups, the respective and complementary roles of PROMs and CROMs in the assessment of postoperative recovery in the context of orthopaedic rehabilitation were evaluated.

## 2. Materials and Methods

### 2.1. Aim, Design, and Setting of the Study

This cohort study was conducted to analyse changes and correlations between CROMs and PROMs at the beginning (t1) and end (t2) of orthopaedic rehabilitation (<19 days in-between) to describe the measurement properties of CROMs and PROMs in the recovery process after knee arthroplasty and hip arthroplasty, and to compare these. We applied a distribution-based approach that dynamically adjusts the final discharge score by systematically accounting for the individual improvement observed throughout the rehabilitation process. This method ensures a more accurate and individualised assessment of rehabilitation success, referred to as the ‘stratified performance score T2D’ [36,39]. This alternative metric adjusts discharge health status (t2) by observed changes in status over time (t2 − t1). In this approach, the initial values (t1) are taken into account in the valuation, while considering mathematical coupling between t1 and t2 − t1 (c.f., Section 2.2.3).

In Austria, all total joint replacement (TJR) patients are offered inpatient rehabilitation for 21 days [40]. According to the WHO definition, this rehabilitation includes a phase II follow-up treatment or post-acute therapy in specialised rehabilitation centres. The patient must fulfil three conditions to be eligible for medical rehabilitation: they must demonstrate a need for rehabilitation, be suitable for rehabilitation (motivated and able to participate in rehabilitation care), and be able to achieve the specific goal of rehabilitation care within a certain time frame [41]. The mean period between acute care (surgery) and rehabilitation for orthopaedic patients who have undergone a surgery of the knee or hip is around 10 weeks [37].

This study was conducted at the Rehabilitation Center Kitzbühel, a specialised orthopaedic facility in Austria, and included patients who had undergone total hip or knee arthroplasty. All participants completed a structured 21-day inpatient programme based on national standards for musculoskeletal rehabilitation. The programme featured two daily physiotherapy sessions (30–45 min), combining individual and group exercises to improve mobility, strength, proprioception, and gait. Additional components included functional training, occupational therapy when indicated, and standardised education on joint protection, pain management, and physical activity. Therapy intensity was adjusted to individual capacity, while the overall structure and frequency of interventions remained consistent. All treatments were delivered by licensed professionals under interdisciplinary supervision according to institutional guidelines.

The patients were fully informed of the data content and purpose of its use and had given their written informed consent for its scientific use. The Ethics Committee of the Medical University of Innsbruck approved the study protocol on 23 August 2019 (Ref: EC Nr: 1158/2019). It was retrospectively entered into the German Clinical Trials Register on 14 August 2020 (DRKS, registration number: DRKS00022854).

### 2.2. Outcome Measures

At the beginning (t1) and the end of rehabilitation (t2), clinician-reported data (CROMs) and self-reported data (PROMs) were collected.

#### 2.2.1. Clinical Anthropometric Measures and Clinician-Reported Outcome Measures (CROMs)

Before the study started, the clinicians and therapists involved in the outcome measurements participated in a standardised training course on data collection to ensure that valid, reliable, and reproducible ROM and TUG data were collected. Experienced therapists used a conventional goniometer to measure the range of motion (ROM) of the knee and hip joints to ensure a high reliability for the longitudinal assessments. ROM calculations were based on reference values representing the range of motion as a percentage of the generally accepted normal range of the American Academy of Orthopaedic Surgeons (AAOS) active range of motion score [42,43]. Functional mobility was assessed using the TUG test. For the statistical analysis, TUG and ROM data were combined, and a mean value was calculated using the *z*-transformation to obtain a mean CROM score (mean CROMs).

#### 2.2.2. Patient-Reported Outcome Measures (PROMs)

PROMs were recorded using the following instruments: WOMAC, NPRS, HAQ, and EQ-5D (TTO score and EQ-VAS) [44]. A shortened version of the HAQ, the HAQ-DI, was used in this study [23]. This questionnaire comprises 20 items divided into eight categories, which enables a comprehensive assessment of functional activities. For the statistical analysis, all PROMs were combined, and a mean score was calculated using the *z*-transformation to obtain the mean PROM score (mean PROMs). An overall medical quality index (MQOidx) was calculated from the mean value of the individual PROMs and the mean value of the TUG and ROM (mean CROMs).

#### 2.2.3. T2D Performance Scores

Using objective measures of joint mobility can lead to ceiling effects, as some patients already have good scores or reach successful endpoints quickly. For example, if a patient has good knee mobility when starting rehabilitation or can perform the TUG in an acceptable period, a large increase is not expected during rehabilitation. In this case, the patient’s overall performance (healing success) may be good, but only a small increase (improvement) is observed descriptively. This is then interpreted as a low treatment effect or even failure. Therefore, to assess patient performance based on the individual scores, a specific method was used to reflect the fact that the change in scores depends on the patient’s initial functional status [36]. When preoperative scores are not available to assess postoperative progress, the simple formula t2 + (t2 − t1) best reflects performance and takes into account the functional status at the end of rehabilitation and improvements (changes from t1 to t2) [39]. It is possible to interpret ‘performance scores’ using a distribution-based approach in which t2 + (t2 − t1) are transformed into standardised scores with *z*-transformation or percentiles.

### 2.3. Statistical Analysis

SPSS Statistics for Windows (Version 27.0. Armonk, NY, USA: IBM Corp) was used for data analysis. For each outcome measure, score differences (Δ) between the beginning (t1, pre-test score) and the end (t2, post-test score) of rehabilitation were calculated and tested for significant changes using *t*-tests. For multiple comparisons, 2 × 2 MANOVA for repeated measurements was used. *Z*-values and effect sizes for within-subjects designs were calculated (Cohen’s *d*_z_ and partial eta-squared, η_p_^2^). Effect sizes were interpreted according to Cohen (Cohen, 1977) [45].

By means of *z*-standardisation, differently scaled quantities were summarised, and the changes were uniformly quantified for the mean of PROMs and the mean of CROMs. A value of 50% (median) or a *z*-value of zero corresponded to the mean of admission (t1) and discharge (t2) data for the sample. A *z*-difference of zero ± 0.20 represents no significant changes from t1 to t2. Negative *z*-differences (SMD) correspond to an improvement in the outcome measure. Changes from t1 to t2 are revealed by the number of patients (%) that could be improved in statistically relevant ways (categorical presentation). The threshold used was an average *z*-difference (SMD) of >0.20.

Correlations between CROMs and PROMs were determined using Spearman’s rank correlation coefficients (ρ), as not all data met the normality assumption and linear regression models for both t1 and t2 scores.

The difference between the percentiles of PROMs and CROMs was calculated to show the level of consistency. Performance scores (percentiles) for each outcome measure were classified as highly consistent (within one/same tertile), moderately consistent (if the scores ranged between one and two tertiles), or as poorly consistent/discrepant (more than two tertiles of difference between performance scores). By chance, this would result in an equivalence of 33.3% in each category if no correlation existed between the different measured outcomes.

## 3. Results

Between January and December 2018, a cohort of 717 patients completed the 21-day rehabilitation programme. Of these, 409 patients (34.2% male, *n* = 140) had undergone total knee arthroplasty, while 308 patients (36.3% male, *n* = 112) had undergone total hip arthroplasty. The mean age (±SD) in the knee group was 68.3 ± 9.3 years and in the hip group, 68.1 ± 10.6 years. All patients completed the full three-week rehabilitation programme. The average duration of the individual therapy received was 461.4 ± 109.9 min in the knee group and 468.0 ± 100.0 min in the hip group.

Table 1 summarises the changes in the scores and effect sizes in both groups (main effect time, all *p* < 0.001; η_p_^2^ multivariate = 0.694). Large effect sizes (Cohen’s *d*_z_ > 0.97) for CROMs (ROM and TUG) were observed in both groups. Medium effect sizes (*d* ≥ 0.65) were observed for the WOMAC (pain, function, and total score) and NPRS in the knee group and for the HAQ and the WOMAC (function and total score) in the hip group.

The overall Medical Outcome Quality (MQOidx; mean value from PROMs and CROMs) improved in 87.7% of patients by the end of rehabilitation (cut-off: *z*-difference (t2 − t1) < −0.20). In 9.5%, the status remained unchanged, while 2.8% showed a deterioration (>0.20). Significant baseline differences (t1) existed for NPRS, HAQ, WOMAC (pain, stiffness, total score), and ROM (*p* < 0.05; η_p_^2^ = 0.183). CROMs responded more strongly than PROMs: 89.9% of patients improved (knee: 92.4%, hip: 86.4%), and 10.2% remained unchanged, showing no deterioration. In contrast, 71.3% of patients in the PROMs improved, 15.6% remained stable, and 13.1% deteriorated (knee: 12.2%, hip: 14.3%).

Table 2 summarises the correlations between CROMs and PROMs at the beginning (t1) and end (t2) of rehabilitation. The observed correlations and linear regression models were similar at the different time points. In the knee group, only the TUG showed a clear correlation with the HAQ at the beginning (ρ = 0.54) and at the end of rehabilitation (ρ = 0.46). Overall, the correlations between the PROMs and CROMs were less pronounced in this group, especially for t2, where a linear regression between all individual PROMs (EQ-5D, NPRS, HAQ, and WOMAC) and the mean CROMs (TUG and ROM) yielded an *R*^2^ of 0.29 (t1) and 0.19 (t2). Compared to the hip group, a strong positive correlation was found between the HAQ and the TUG [t1: ρ = 0.65; t2: ρ = 0.63] as well as a moderate negative correlation between the HAQ and the ROM [t1: ρ = −0.45; t2: ρ = −0.46]. The WOMAC also showed a strong positive correlation with the TUG (ρ ≥ 0.52). The linear regression between all individual PROMs and the mean CROMs resulted in an *R*^2^ of 0.34 (t1) and 0.39 (t2) for the hip group. Similar associations (cf. regression coefficients) can be observed in patients with total hip arthroplasty, as the multiple regression model shows for t1 CROM_HIP_ = −0.007*EQ-VAS + −0.048*EQ-5D TTO + −0.119*NPRS + 0.456*HAQ + 0.184*WOMAC (*R*^2^ = 0.341, *p* = 0.000 **, *c* = −0.348) and for t2 CROM_HIP_ = 0.039*EQ-VAS + −0.131*EQ-5D TTO + −0.068*NPRS + 0.424*HAQ + 0.189*WOMAC (*R*^2^ = 0.385, *p* = 0.000 **, *c* = 0.083).

**Figure 1:**
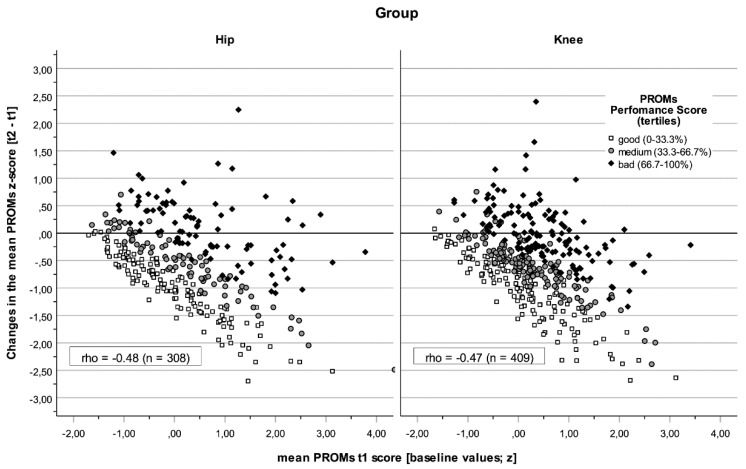
The performance of patients with total knee or hip arthroplasty in PROMs. Dots represent the mean PROMs score at the beginning of rehabilitation (*x*-axis) and their changes (*y*-axis). Classified performance score markers (t2 + Δ) based on normal scores (tertiles); The baseline scores (t1) were more homogeneous in the knee group (left-hand panel; *SD* = 0.93) than in the hip group (right-hand panel; SD = 1.10; Levene Test *p* = 0.001).

**Figure 2:**
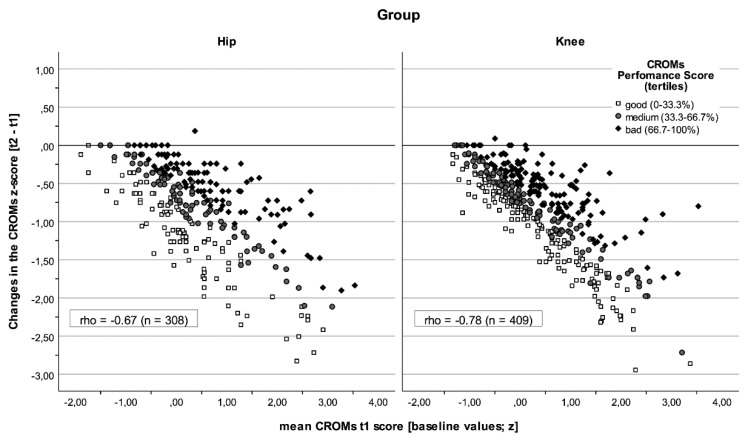
The performance of patients with total knee or hip arthroplasty in CROMs. Dots represent the mean CROM score at the beginning of rehabilitation and its changes. Classified performance score markers based on normal scores (tertiles). The standard deviation between groups was more homogeneous in CROM-changes for the knee group (SD Knee = 0.57 vs. *SD* Hip = 0.69, *p* = 0.048). The hip group (left-hand panel) showed a weaker correlation (ρ = −0.67) between output value (t1) and its change.

The behaviours of the PROMs and CROMs at the beginning of rehabilitation and in relation to the change are shown in Figure 1 and Figure 2. A general trend was observed that the values improved throughout rehabilitation. Some patients’ results worsened significantly as reflected by PROMs but not by CROMs in both groups (see also Table 1 and Table 2). Two effects were observed. First, the differences between the groups were less pronounced; the correlations were similar, but a stronger relationship was seen between the baseline values and their changes in CROMs for the knee group (ρ knee = −0.78 vs. ρ hip = −0.67, *p* = 0.002). Second, PROM baseline scores were more homogeneous in the knee group (Levene Test *p* = 0.001), as were changes in CROMs (*p* = 0.048).

## 4. Discussion

In this observational cohort study, we investigated changes in functional outcomes during rehabilitation after knee arthroplasty and hip arthroplasty, particularly focusing on the measurement properties and responsiveness of PROMs and CROMs. Our results show that CROMs have larger effect sizes than PROMs, indicating that these objective testing procedures are more sensitive for capturing functional improvements. While significant functional improvement was achieved in the knee and the hip group, PROM scores worsened in 13.1% of patients. This could indicate different subjective experiences of functional improvement or a discrepancy between the objective and subjective assessments of rehabilitation progress. The stability of the correlations over time and between the patient groups (Table 2) emphasises the methodological reliability of the assessments used. The similarity of the results obtained for knee and hip patients speaks in favour of the robust applicability of the investigated measurement instruments in both groups.

Correlations between CROMs and PROMs were most pronounced for TUG and HAQ scores. Weaker correlations could be observed between the ROM and PROMs. Therefore, the TUG test results reflect comparable PROM functions and limitations in patients undergoing hip or knee arthroplasty more accurately than the ROM. It should be noted that TUG and ROM capture different functions or modalities. This is especially true for knee patients, where the correlation between ROM and TUG is remarkably low (ρ = −0.21). However, in more than half of the cases, this seems to have had a lower impact on the consistency classification of the performance evaluation, whereby the level of agreement between the individual PROMs and combined CROMs is similar in both patient groups.

The moderate correlations between PROMs and CROMs observed in this study are supported by the literature [37,38,46,47]. Performance-based tests of physical function (CROMs) offer healthcare professionals the opportunity to objectively record what patients can actually do, while PROMs are based on patients’ subjective assessments of their abilities. These methodological differences explain the frequently observed moderate correlations between the two measurement approaches [5,13,14,31,32,33,34,35,36]. The TUG is one of the performance-based tests recommended by the Osteoarthritis Research Society International (OARSI) [17] and provides a means of practically assessing functional mobility. Our results show that the TUG reliably represents patients’ mobility abilities and correlates closely with PROMs of functional limitation, particularly with the HAQ. This could be due to the test design, which integrates everyday movement patterns [48]. In contrast, the ROM measurements correlated less strongly with PROMs, suggesting that this biomechanical measure allows only limited conclusions to be drawn about perceived functional limitations.

Another important aspect is the temporal development of the correlations between PROMs and CROMs. The results of the study show that CROMs, in particular ROM and TUG, have a high sensitivity for detecting functional improvement in the acute postoperative period, whereas PROMs are more indicative of long-term clinical outcomes [19,49,50]. This change in sensitivity may occur because objective functional improvement is more easily measurable immediately after surgery, while subjectively perceived improvement emerges gradually as the patient adapts to the new joint replacement. Other studies have shown that postoperative changes are particularly significant in the subacute phase (approximately one month after arthroplasty). Comparisons of the results in this period show contradictory or even reversed courses of functional improvement [51,52]. This emphasises the need for a targeted temporal adjustment of the assessment instruments in the recovery process. While objective measures provide a more reliable basis for assessing functional improvement in the early phases of rehabilitation, PROMs may play a greater role in later phases as they reflect the long-term integration of functional improvements into daily life. Future research should investigate how the optimal timing for the use of these tools can be defined to enable more precise course control and tailored treatment decisions.

There are obvious interdependencies between patient-reported outcomes and performance measures, but these are used to evaluate different outcomes and to assess complementary, important modalities in orthopaedic rehabilitation. Our finding is consistent with those of Stratford et al. [53], who proposed that self-reported outcomes and performance measures can be used to assess different aspects of physical function. These authors concluded that self-report measures provide information about the experience of performing the task, while performance measures contain information about the ability to perform the task. A gap often occurs between personal intention and patient behaviour, e.g., between high self-efficacy and a positive attitude towards physical activity, although this does not necessarily lead to increased physical activity [54]. Due to the tight schedule and the intensive rehabilitation process, this effect could be directly noticed and distinguished in our patients in this study. We observed that better mobility was associated with an increase in all reported endpoints, but the relationships between the different measurement modalities were not always clear. These findings agree with those of Melzer et al. [55], who reported detecting poor-to-moderate associations between performance-based measures and self-reported functional status in older patients. The discrepancy between objective functional improvements and subjective complaints can be explained by several mechanisms. In addition to the inherent subjectivity of patient-reported outcomes, neurophysiological processes [56], motor adaptation strategies [57], and sociocultural factors [58] also play a decisive role. Central nervous sensitisation and altered pain inhibition can lead to patients continuing to experience pain despite objective functional improvement. Neurotransmitters such as serotonin and dopamine influence pain processing and could explain the difference between objective measurements and subjective perception [56]. In addition, compensatory movement patterns can contribute to the discrepancy between objective and subjective results. Modified postures or altered gait patterns can maintain or even improve functionality without reducing the perception of pain [57]. This emphasises that a purely objective assessment of functional improvement may be incomplete and may not adequately reflect the subjective burden of patients.

The study results emphasise the need for a multidimensional assessment approach in musculoskeletal rehabilitation. After surgical interventions such as knee or hip arthroplasty, the combination of objective and subjective measurement methods enables not only a more comprehensive assessment of functional improvements but also the identification of neurophysiological regulatory mechanisms. This knowledge can be used specifically to optimise rehabilitation strategies, promote a more sustainable recovery of mobility, and determine the optimal time to use these assessment tools.

Compared to international studies, such as that of Monselli et al. [59], which also investigated postoperative rehabilitation protocols after hip arthroscopy, our results confirm the consensus on the effectiveness of a stepwise rehabilitation approach. The current literature underlines the need for standardised yet flexible and adaptable guidelines that take into account both individual needs and the patient’s activity level. A multidimensional assessment approach that integrates clinical and functional parameters is therefore essential in order to holistically record and specifically optimise rehabilitation outcomes. Our study provides practical evidence by analysing comparative data on PROMs and CROMs in the knee compared to the hip group. The results underline the clinical benefits of the combined use of both methods, as they enable a differentiated assessment of functional progress and thus support individually optimised treatment planning.

### 4.1. Limitations

This study has some methodological limitations that should be considered when interpreting the results. First, the analysis is based on a cohort from a single specialised rehabilitation centre, which may limit the generalisability of the results to other settings, and especially outpatient rehabilitation or international comparison groups. In addition, rehabilitation in an inpatient setting can influence the recruitment of patients through clinical and socioeconomic factors and thus further distort the results. Second, PROMs can be influenced by subjective factors such as pain perception, mental state, memory, or patient expectations, which can lead to bias. Therefore, the observed discrepancy between PROMs and CROMs could be due to methodological differences and/or inter-individual differences in the patients’ perceptions and self-assessments. Third, a relatively short period of three weeks may not have been sufficient for the clinical condition to reach a stable baseline of improvement. Future studies should consider a longer follow-up period to better capture the long-term effects of rehabilitation and provide a more comprehensive understanding of recovery progress. Finally, we did not stratify the analysis by demographic or clinical subgroups such as age, comorbidities, or preoperative functional levels. Such subgroup analyses could help to identify conditions under which PROMs or CROMs, or their interaction, may be more informative and guide more tailored outcome assessments in clinical practice.

### 4.2. Clinical Relevance

The results of this study have important clinical implications for rehabilitation after knee and hip arthroplasty. The observed improvements in both PROMs and CROMs confirm the effectiveness of structured rehabilitation programmes and support their continued use in outcome monitoring. The stronger correlation between the TUG test and the PROMs compared to the CROMs suggests that mobility-based performance measures are more consistent with patients’ subjectively perceived function. At the same time, the discrepancy between PROMs and CROMs highlights the need for a multidimensional assessment approach that integrates both subjective and objective parameters. While PROMs reflect the individual perception of pain and stress, CROMs capture objective functional progress. An exclusive focus on PROMs carries the risk of overlooking significant functional improvements. Therefore, clinicians and therapists should systematically include CROMs in their assessment procedures, especially in Phase II of the rehabilitation process. This integrative approach enables early detection of discrepancies, targeted adaptation of therapy, and the development of more individualised treatment strategies. The combined use of PROMs and CROMs can also improve treatment adherence and patient satisfaction, as well as provide a more informed assessment of short- and long-term outcomes.

## 5. Conclusions

This study demonstrates that PROMs and CROMs capture distinct yet complementary aspects of recovery after knee and hip arthroplasty and that they should not be used interchangeably. While CROMs are more sensitive to short-term functional gains, PROMs reflect the patient’s longer-term perception of recovery. The modest correlation between the two suggests that relying on either alone may lead to incomplete or misleading assessments. The findings support maintaining CROMs in routine clinical practice despite their resource demands. The integration of both types of measures facilitates more precise monitoring of progress, timely adjustment of rehabilitation strategies, and improved alignment between patient needs and clinical decision-making. Future research should focus on refining the timing and choice of outcome measures, as well as their interrelationships, to improve the prediction of long-term rehabilitation success.

## Figures and Tables

**Table 1:** PROMs and CROMs in the knee and hip group. Quality-of-outcome measures were assessed and documented in the discharge report at the beginning (t1) and end (t2) of the 21-day inpatient rehabilitation programme. Statistically significant improvements were observed for all outcome measures in the knee group (all *p* < 0.001; η_p_^2^ _multivariate_ = 0.745) and the hip group (all *p* < 0.001; η_p_^2^ _multivariate_ = 0.670).

TOTAL (*n* = 717)		t1 (Mean ± SD)			t2 (Mean ± SD)			Changes (Δ) (Mean ± SD)			Cohen’s *d_z_*
KNEE (*n* = 409)											
PROMs	EQ-5D Health (EQ-VAS)	64.89	±	18.03	69.35	±	22.24	4.47	±	22.64	0.20
	EQ-5D TTO	0.81	±	0.17	0.88	±	0.11	0.06	±	0.20	0.42
	NPRS	4.25	±	1.90	3.05	±	1.83	−1.21	±	1.70	0.69
	HAQ	0.74	±	0.45	0.56	±	0.43	−0.18	±	0.35	0.53
	WOMAC total score	75.42	±	40.03	49.37	±	35.20	−26.04	±	30.00	0.88
	**Mean PROMs [** ***z*** **]**	**0.31**	**±**	**0.93**	**−0.25**	**±**	**0.86**	**−0.56**	**±**	**0.70**	**0.81**
CROMs	ROM [%]	62.77	±	11.85	72.78	±	7.85	10.02	±	7.10	1.41
	TUG [sec]	12.30	±	4.97	9.51	±	3.45	−2.79	±	2.80	1.01
	**Mean CROMs [*z*]**	**0.40**	**±**	**0.99**	**−0.46**	**±**	**0.66**	**−0.86**	**±**	**0.60**	**1.49**
HIP (*n* = 308)											
PROMs	EQ-5D Health (EQ-VAS)	64.29	±	18.50	73.11	±	20.80	8.83	±	22.00	0.40
	EQ-5D TTO	0.82	±	0.17	0.86	±	0.15	0.05	±	0.20	0.31
	NPRS	3.41	±	2.10	2.61	±	1.94	−0.80	±	1.80	0.44
	HAQ	0.95	±	0.53	0.66	±	0.52	−0.28	±	0.35	0.80
	WOMAC total score	66.21	±	43.60	43.11	±	38.30	−23.1	±	32.00	0.73
	**Mean PROMs [** ***z*** **]**	**0.24**	**±**	**1.08**	**−0.33**	**±**	**1.00**	**−0.57**	**±**	**0.80**	**0.76**
CROMs	ROM [%]	59.64	±	11.60	68.64	±	9.40	9.00	±	8.70	1.03
	TUG [sec]	13.15	±	6.00	9.85	±	3.77	−3.30	±	3.40	0.97
	**Mean CROMs [*z*]**	**0.47**	**±**	**1.14**	**−0.39**	**±**	**0.79**	**−0.86**	**±**	**0.70**	**1.24**

Δ = difference (t2–t1); bold summarised mean value of PROMs or CROMs; EQ-5D = European Quality of Life-5 Dimensions 5 levels; HAQ = Health Assessment Questionnaire; NPRS = Numeric Pain Rating Scale; WOMAC = Western Ontario and McMaster Universities Osteoarthritis Index; ROM = range of motion; TUG = Timed Up and Go.

**Table 2:** Correlations between PROMs and CROMs in t1 and t2 for the knee group. The correlations and linear regression models were similar at the different time points. The associations between PROMs and the TUG were more pronounced than those between PROMs and the ROM. The strongest relationship to CROMs could be found with the HAQ. The correlation for changes between averaged PROMs and CROMs is 0.19 and ρ = 0.17 for the performance score.

		t1			t2		
KNEE		ROM [%]	TUG [sec]	Mean CROMs [*z*]	ROM [%]	TUG [sec]	Mean CROMs [*z*]
PROMs	EQ-5D Health (EQ-VAS)	0.12 *	−0.22 **	−0.21 **	0.16 **	−0.16 **	−0.20 **
	EQ-5D TTO	0.13 **	−0.26 **	−0.23 **	0.13 **	−0.28 **	−0.25 **
	NPRS	−0.19 **	0.23 **	0.26 **	−0.15 **	0.25 **	0.23 **
	HAQ	−0.30 **	0.54 **	0.49 **	−0.19 **	0.46 **	0.39 **
	WOMAC total score	−0.30 **	0.30 **	0.36 **	−0.20 **	0.27 **	0.29 **
	**Mean PROMs [*z*]**	−0.28 **	0.40 **	0.41 **	−0.22 **	0.35 **	0.35 **
CROMs	ROM [%]	--	−0.33 **	−0.83 **	--	−0.21 **	−0.80 **
	TUG [sec]	−0.33 **	--	0.77 **	−0.20 **	--	0.70 **
	**Mean CROMs [*z*]**	−0.83 **	0.77 **	**--**	−0.80 **	0.70 **	**--**

Spearman correlations (ρ) for t1/t2 (*n* = 409); bold = summarised mean PROMs/CROMs. t1 CROM_KNEE_ = −0.024*EQ-VAS + 0.052*EQ-5D TTO + 0.071*NPRS + 0.480*HAQ + 0.066*WOMAC (*R*^2^ = 0.290, *p* = 0.000 **, *c* = −0.839). t2 CROM_KNEE_ = −0.049*EQ-VAS + −0.092*EQ-5D TTO + 0.040*NPRS + 0.320*HAQ + 0.013*WOMAC (*R*^2^ = 0.191, *p* = 0.000 **, *c* = 0.402). Test–retest reliability (Pearson correlation t1 to t2): PROMs = 0.38 to 0.81, *p* < 0.001; CROMs = 0.81 to 0.84, *p* < 0.001. Spearman’s rank correlation coefficients (ρ); baseline (t1). Study end (t2); EQ-5D = European Quality of Life-5 Dimensions 5 levels (Ludwig et al., 2018) [44]; HAQ = Health Assessment Questionnaire; NPRS = Numeric Pain Rating Scale; WOMAC NRS = Western Ontario and McMaster Universities Osteoarthritis Index, significances are marked with an asterisk: * *p* < 0.05; ** *p* < 0.01.

## Data Availability

The research data supporting this publication are stored in our institutional digital data repository for published research, accessible via https://creed.lbg.ac.at (accessed on 3 March 2025). The data sets analysed in this manuscript are not publicly available due to ethical and legal restrictions, as they contain potentially identifying and sensitive patient information. However, pseudonymised data sets have been created for the purpose of re-use and are also accessible at creed.lbg.ac.at. Requests for access to anonymised data sets should be directed to the corresponding author (V.G.).
